# Supplementary material for: Three-dimensional observations of the electric field distribution of variable frequency microwaves, and scaling-up organic syntheses
Source: Commun Chem. 2023 Nov 29;6:261. doi: 10.1038/s42004-023-01062-6 (PMC10687222; doi:10.1038/s42004-023-01062-6)
Supplement: Supplementary file 2 — Supplementary Information [file 42004_2023_1062_MOESM2_ESM.pdf]

Three-dimensional Observations of the Electric Field Distribution of Variable Frequency Microwaves, and Scaling-up Organic Syntheses

Satoshi Horikoshi *[1], Haruka Mura [1], Nick Serpone [2]

[1] *Department of Materials and Life Sciences, Faculty of Science and Technology, Sophia University, 7-1 Kioicho, Chiyodaku, Tokyo 102-8554, Japan*
ORCID(SH): 0000-0001-6588-5821

[2] *PhotoGreen Laboratory, Dipartimento di Chimica, Università di Pavia, Via Taramelli 12, Pavia 27100, Italy*
ORCID(NS): 0000-0003-0236-9795

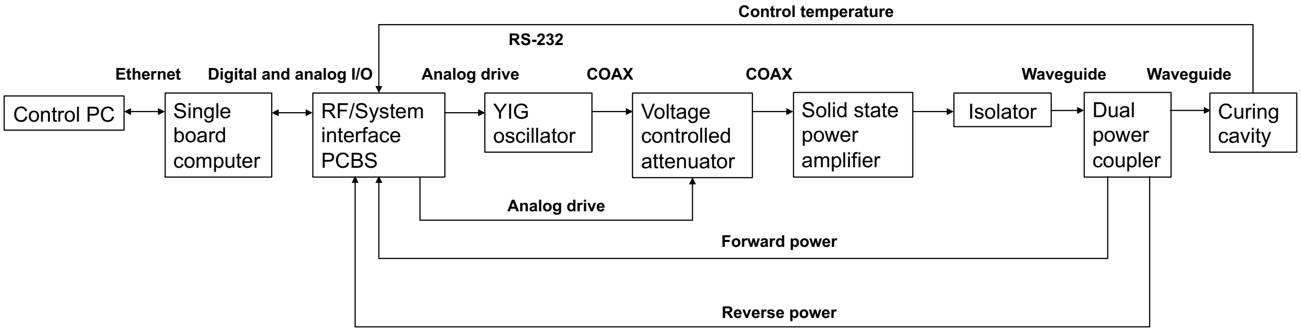

Fig. SI-1: Block diagram of the VFM control system in the VariWave equipment.

Three-dimensional Observations of the Electric Field Distribution of Variable Frequency Microwaves, and Scaling-up Organic Syntheses

Satoshi Horikoshi *[1], Haruka Mura [1], Nick Serpone [2]

[1] *Department of Materials and Life Sciences, Faculty of Science and Technology, Sophia University, 7-1 Kioicho, Chiyodaku, Tokyo 102-8554, Japan*
ORCID(SH): 0000-0001-6588-5821

[2] *PhotoGreen Laboratory, Dipartimento di Chimica, Università di Pavia, Via Taramelli 12, Pavia 27100, Italy*
ORCID(NS): 0000-0003-0236-9795

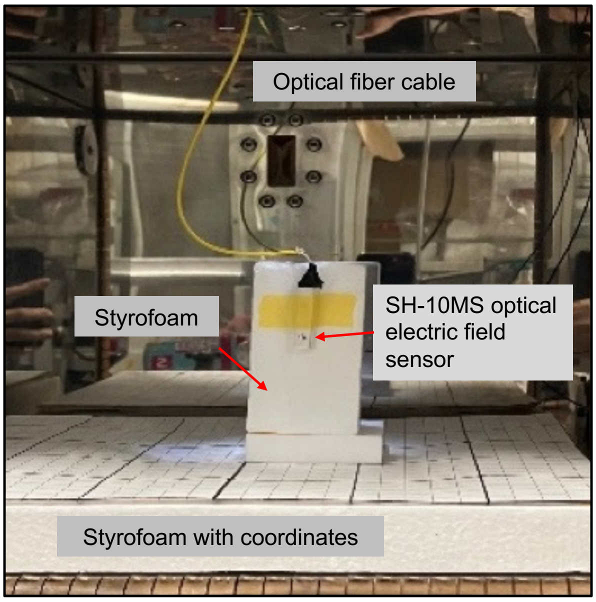

Fig. SI-2: Photograph of the SH-10MS optical electric field sensor fixed with Styrofoam at the measurement position in the cavity of the VariWave system.
