# Supplementary material for: Three-dimensional observations of the electric field distribution of variable frequency microwaves, and scaling-up organic syntheses
Source: Commun Chem. 2023 Nov 29;6:261. doi: 10.1038/s42004-023-01062-6 (PMC10687222; doi:10.1038/s42004-023-01062-6)
Supplement: Supplementary file 3 — Description of Additional Supplementary Files [file 42004_2023_1062_MOESM3_ESM.pdf]

Description of Additional Supplementary Files

File name: Supplementary Data 1
Description: The source data behind the graphs in the figures.
